# Effect of Block Freeze Concentration on Bioactive Compound Content and Antioxidant Capacity When Applied to Peppermint (*Mentha Piperita* L.) Infusion

**DOI:** 10.3390/antiox14020129

**Published:** 2025-01-23

**Authors:** Indira Pérez-Bermúdez, Alison Castillo-Suero, Constanza Jara-Leiva, Axel Cortés-Valdivia, Karol Rojas-Rojas, Vivian García-Rojas, Mauricio Opazo-Navarrete, María Guerra-Valle, Guillermo Petzold, Patricio Orellana-Palma

**Affiliations:** 1 Grupo de Crioconcentración de Alimentos y Procesos Relacionados, Departamento de Ingeniería en Alimentos, Facultad de Ciencias de la Salud y de los Alimentos, Universidad del Bío-Bío, Campus Fernando May, Av. Andrés Bello 720, Chillán 3780000, Chile; indira.perez2001@alumnos.ubiobio.cl (I.P.-B.); vivian.garcia@userena.cl (V.G.-R.); 2 Departamento de Ingeniería en Alimentos, Facultad de Ingeniería, Universidad de La Serena, Campus Andrés Bello, Av. Raúl Bitrán 1305, La Serena 1720010, Chile; alison.castillos@userena.cl (A.C.-S.); cjaral@alumnosuls.cl (C.J.-L.); axel.cortes@userena.cl (A.C.-V.); karol.rojasr@userena.cl (K.R.-R.); 3 Agriaquaculture Nutritional Genomic Center (CGNA), Temuco 4780000, Chile; mauricio.opazo@cgna.cl; 4 Escuela de Nutrición y Dietética, Facultad de Ciencias Para el Cuidado de la Salud, Universidad San Sebastián, Campus Concepción, Lientur 1457, Concepción 4080871, Chile; maria.guerra@uss.cl

**Keywords:** *Mentha piperita* L., infusion, freeze concentration, physicochemical analysis, bioactive compounds, antioxidant capacity

## Abstract

This research aimed to evaluate block freeze concentration (BFC) under different centrifugation conditions using response surface methodology to separate an extract from the ice fraction at three centrifugal-BFC (CBFC) cycles, obtaining in the final cycle a phenolic-rich extract. A Box–Behnken design was applied to optimize centrifugation variables, with efficiency of separation (η) selected as the response variable. The extracts were characterized in terms of physicochemical analysis, total and individual bioactive components, and antioxidant capacity. Optimal conditions (3600 rpm, 16 °C, and 14 min) resulted in η of 82%. Thus, from infusion to final cycle, the solids, total polyphenol and flavonoid contents, and antioxidant capacity exhibited from 1.81 to 6.5% (*w*/*w*) and 2.5 to 8.7 (°Brix), 0.72 to 12.2 mg gallic acid equivalents/mL, 0.83 to 13.7 mg catequin equivalents /mL, 2.8 to 31.2 μmol trolox equivalents/mL and 4.8 to 122.2 μmol trolox equivalents/mL, identifying by high-performance liquid chromatography that kaempferol, *p*-hydroxybenzoic, and transferulic acid presented the highest concentrations. The CBFC process has the potential as a non-thermal concentration process to preserve many bioactive compounds, facilitating the production of concentrated fractions with high biological value, where the extracts obtained by BFC are a novel solution for medicinal, pharmaceutical, and food applications.

## 1. Introduction

For centuries, plants and herbs have attracted attention for their aromatic and therapeutic properties, being used in gastronomy, perfumery, cosmetics, and food areas, highlighting pharmaceutical formulations and nutritional/functional foods in particular [1], since these plants and herbs provide vitamins, minerals, and phytochemicals (phenolic acids, flavonoids, tannins, stilbenes, and anthocyanins, among others, which enable high antioxidant activity) [2]. Thus, these medicinal plant-based products can contribute to protecting cells against oxidative damage, preventing or curing different illnesses such as respiratory and digestive disorders, and reducing the risk of cancer, diabetes, ageing-related diseases, cardiovascular disease, and other degenerative diseases [3,4,5,6,7]. Moreover, these products are cost-effective alternatives that can help reduce the need for synthetic antibiotics and increase life expectancy [8,9]. In this way, different food formats based on medicinal plants can be found in the global market such as powders, tinctures, and poultices, among others [10]. Various technologies have been used to extract biocomponents from plants and herbs for direct consumption or to be used as an ingredient in the production of food products.

In particular, the World Health Organization (WHO) reported that approximately 80% of the world’s population uses herbal medicines in the treatment of various diseases, where over 25% of prescribed medicines are derived from wild plant species [10]. In addition, the worldwide market for herbal products was valued at USD 148.5 billion in 2022, with an estimated worldwide market value of USD 174.49 billion in 2024, with an expected increase to USD 386 billion in 2032, projecting an annual growth rate of 11.2% from 2023 to 2032 [11]. These data suggest that the demand for herbal medicines will continue to escalate in the forthcoming years.

Specifically, peppermint, commonly known as mint, has been recognized as a valuable source of biologically active compounds such as rutin, eugenol, apigenin, α-tocopherol, flavonoids, betaine, carvone, and ascorbic, oleanic, caffeic, rosmarinic and ursolic acids [12,13,14,15], providing anticancer, antineoplastic, antiviral, antihistaminic, and anti-inflammatory applications, adding analgesic, diuretic, hypertension-controlling, and urease-inhibiting properties [16,17,18,19,20,21]. Furthermore, *Mentha* spp. are used in the food industry for their well-known culinary applications, highlighting the tea infusion formulation [22]. Therefore, the use of novel, non-thermal processing technologies is an excellent opportunity to protect bioactive compounds, retain sensory attributes, improve the shelf life, and avoid the loss of heat-sensitive nutritional components and textural and rheological characteristics in the concentrates from the original sample in comparison to the more traditional concentration technologies (evaporation).

Thus, freeze concentration (FC) technology is an interesting alternative to concentrate liquid samples since FC is a novel emerging, cost-effective, and eco-friendly method. Thermal evaporation (TE) and membrane technology (MT) have lower capital requirements, but the operational and maintenance costs are higher than FC [23], whereas TE and MT need additional pretreatment units, energy, materials, and cleaning chemicals to overcome fouling [24]. Between FC methods, for suspension FC has a higher initial investment and capital costs in comparison to BFC [25]. Additionally, FC does not use components that favor phase separation (e.g., ethanol and methanol, among others), helping to ensure that there is no waste after obtaining the concentrate [26].

FC is based on concentration at freezing temperatures, where the frozen sample forms two phases: a porous ice phase (the free water freezes) and a concentrate phase (an extractable liquid) [27]. Thus, FC technology has been successfully applied to concentrate different liquid foods, with excellent retention of polyphenols, anthocyanins, and flavonoids, among others, since the low temperatures do not degrade thermolabile metabolites, producing a concentrate rich in secondary metabolites, retaining bioactive compounds, and presenting a high content of antioxidant activity; i.e., FC maintains the biological activities of the phytochemical constituents, minimizing the loss of volatile compounds, allowing a good preservation of nutritional and organoleptic properties. Moreover, FC generates a concentrate with a high amount of solids, and thus, it is possible to reconstitute the concentrate and consume it at any time just by adding water, helping to use less space and weight at the storage location, which allows economic savings and solving logistical problems for both consumers and large-scale industries [26].

However, to our current knowledge, there are few studies associated with FC applied to herbal infusions of medicinal plants; these have indicated promising results in extract of yerba mate (*Ilex paraguariensis* A. St. Hil.) leaves [28], green tea (*Camellia sinensis*) extract [29], noni (*Morinda citrifolia* L.) tea [30,31], and aqueous extract of guava (*Psidium guajava*) leaves [32], but there are no studies on peppermint infusion.

Therefore, this study sought to evaluate the effects of FC when applied to peppermint infusion. The separation conditions were classified as centrifugation conditions (speed, temperature, and time), and these conditions were optimized using a Box–Behnken design and response surface methodology to maximize the efficiency of separation. The optimal conditions to use were considered at three centrifugal-BFC (CBFC) cycles. Thus, each extract was evaluated in terms of physicochemical properties, bioactive compounds (total polyphenol and flavonoid contents, and HPLC analyses), and antioxidant capacity (DPPH and FRAP assays). Hence, this study also aimed to assess the optimal conditions for obtaining an extract rich in solutes, phenolic compounds, and antioxidant capacity.

## 2. Materials and Methods

### 2.1. Materials

Peppermint (*Mentha piperita* L.) leaves were purchased in a store located in Concepción, Chile, collected in a sealed glass bottle, and transported to the Universidad del Bío-Bío (Chillán, Chile). There, the leaves were cleaned completely, exposed to sunlight for one day, and then, dried at 80 °C for 2 h using a hot air oven (Memmert UF55m, GmbH + Co. KG, Buechenbach, Germany). Then, the dried leaves were collected in a sealed glass bottle and stored at room temperature (≈22 °C) until the preparation of the infusion.

For a polyphenolic profile using high-performance liquid chromatography (HPLC), phenolic standards, including chlorogenic acid (≤100 purity, CAS:327-97-9, Sigma-Aldrich Inc., St. Louis, MO, USA), gallic acid (≥99 purity, CAS:5995-86-8, Sigma-Aldrich Inc., St. Louis, MO, USA), protocatechuic acid (≤100 purity, CAS:99-50-3, Sigma-Aldrich Inc., St. Louis, MO, USA), catechin (≥99 purity, CAS:205-825-1, Supelco, Bellefonte, PA, USA), caffeic acid (≥98 purity, CAS:339-31-5, Sigma-Aldrich Inc., St. Louis, MO, USA), vanillic acid (≥98 purity, CAS:121-34-6, PhytoLab, Vestenbergsgreuth, Germany), syringic acid (≥98 purity, CAS:530-57-4, Sigma-Aldrich Inc., St. Louis, MO, USA), *p*-coumaric acid (≥98 purity, CAS:501-98-4, Sigma-Aldrich Inc., St. Louis, MO, USA), ellagic acid (≥95 purity, CAS:208-507-3, Supelco, Bellefonte, PA, USA), transferulic acid (≥99 purity, CAS:537-98-4, Sigma-Aldrich Inc., St. Louis, MO, USA), rutin (≥94 purity, CAS:250249-75-3, Supelco, Bellefonte, PA, USA), *p*-hydroxybenzoic acid (≥99 purity, CAS:99-96-7, Sigma-Aldrich Inc., St. Louis, MO, USA), myricetin (≥98 purity, CAS:529-44-2, Supelco, Bellefonte, PA, USA), quercetin (≥98 purity, CAS:482-35-9, Supelco, Bellefonte, PA, USA), and kaempferol (≥99 purity, CAS:520-18-3, Supelco, Bellefonte, PA, USA) were used to construct the calibration curve for the identification and quantification of the phenolic compounds in the peppermint infusion and extracts obtained by the BFC method.

The Folin–Ciocalteu phenol reagent, 1,1-diphenyl-2-picrylhydrazyl (DPPH), 2,4,6-tris(2-pyridyl)-s-triazine (TPTZ), 6-hydroxy-2,5,7,8-tetramethylchroman-2-carboxylic acid (Trolox), gallic acid, and catechin were purchased from Sigma-Aldrich Inc. (Burlington, MA, USA). Methanol (≥99.5% purity), sodium carbonate (Na_2_CO_3_), sodium hydroxide (NaOH), sodium nitrite (NaNO_2_), aluminum chloride (AlCl_3_), acetic acid (CH_3_;COOH), acetonitrile (CH_3_CN), sodium acetate (CH_3_COONa), hydrochloric acid (HCl), and iron (III) chloride hexahydrate (FeCl_3_∙6H_2_O) were purchased from Winkler Ltda (Santiago, Chile). The analytical grade of these reagents was used. Ultrapure water was obtained from a Milli-Q Plus water system (Millipore, Bedford, MA, USA).

### 2.2. Preparation of Aqueous Solution (Infusion)

The infusion was obtained using the methodology proposed by Voung et al. [33]. Peppermint leaves (1 g of intact leaves) were placed in a conical flask with 100 mL of distilled water, heated, and kept at 80 ± 1 °C for 30 min in a temperature-controlled orbital shaker. Then, the leaves were separated from the infusion through filtration using qualitative filter paper (125 mm diameter, Unifil^®^, Zilquimica Products Ltd., São Paulo, Brazil), the infusion was cooled at room temperature (≈22 °C), and different analyses (physicochemical analysis, identification of bioactive components, and evaluation of antioxidant capacity) were carried out immediately after preparation of this infusion. The remaining infusion sample was used for the preparation of extracts by CBFC.

### 2.3. BFC Assisted by Centrifugation Procedure

**Figure 1:**
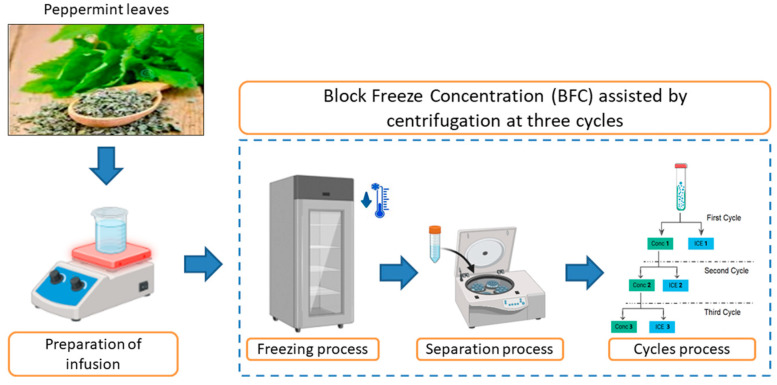
Schematic illustration of the centrifugal-BFC procedure used in this study.

The BFC procedure was performed using the protocol outlined by Orellana-Palma et al. [34]. The infusion (45 mL in Falcon tubes isolated with thermal polystyrene to generate an axial heat transfer) was frozen (−20 °C/12 h) in a static freezer (280, M and S Consul, Sao Paulo, Brazil). Then, the frozen samples were transferred to a centrifuge (Eppendorf 5430R, Hamburg, Germany) to induce the separation of the concentrated fraction (C_f_) from the ice fraction (I_f_) using different centrifugation conditions (speed (CS), temperature (CT), and time (Ct)) (process optimization). Hence, with the best conditions from the optimization, the CBFC procedure was conducted in three cycles, in which the C_f_ from cycle 1 (C_1_) was collected and used as the solution for the next cycle (the concentrate was frozen under the same freezing and separation conditions), and the C_f_ from cycle 2 (C_2_) was used for the third cycle (C_3_). Thus, the characterization in terms of physicochemical properties, identification of bioactive components, and evaluation of antioxidant capacity was made in each extract obtained by this centrifugal-BFC process. Figure 1 shows the complete CBFC procedure.

### 2.4. Box–Behnken Design (BBD) and Response Surface Methodology (RSM)

A BBD was established by RSM to optimize the conditions in the separation process of the C_f_ from the I_f_, using three independent variables, with three equally spaced levels, highlighting CS (2500, 3500, and 4500 rpm), CT (10, 15, and 20 °C), and Ct (10, 20, and 30 min). The response variable (dependent variable) was efficiency of separation (η). Thus, the best combination allows obtaining the highest concentration of solutes, bioactive compounds, and antioxidant capacity values in the C_f_ in each cycle. Table 1 shows the BBD, with independent variables codified at three levels, i.e., (−1) low, (0) medium, and (+1) high.

The relationship between the independent variables and the response was investigated using a second-order polynomial model as shown in Equation (1):

(1) $$Y=\beta_{0}+\sum_{i=1}^{k}\beta_{i}x_{i}+\sum_{i=1}^{k}\beta_{ii}x_{ii}^{2}+\sum_{i=1}^{k-1}\sum_{i=1}^{k}\beta_{ij}x_{i}x_{j}+e_{i}$$

where *Y* is the predicted response, *β*_0_ is the model intercept coefficient, *β_i_* is the linear coefficient (*i* = 1, 2, and 3), *β_ii_* is the quadratic coefficient (*i* = 1, 2, and 3), and *β_ij_* is the cross-product coefficient (*i* = 1, 2, and 3, and *j* =1, 2, and 3), respectively, *x_i_* and *x_j_* are variables, *k* is the number of independent parameters, and *e_i_* is the error.

### 2.5. Response Variable (Efficiency of Separation, η)

η (Equation (2)) can be defined as the increase in the concentration of the solution relative to the amount of solute remaining in the frozen fraction [35].

(2) $$\eta \left(\%\right)=\left(\frac{C_{f}\;-\;I_{f}}{C_{f}}\right)\times 100$$

where C_f_ and I_f_ are the solutes in the concentrated fraction and ice fraction, respectively.

### 2.6. Physicochemical Analysis

The total solid content (TSC) was determined by measuring weight loss upon drying in an oven at 105 °C for 24 h, and the results were expressed as the percentage weight/weight (% *w*/*w*) [36]. The total soluble solids content (TSSC) was analyzed with a digital refractometer (PAL-1, Atago Co., Tokyo, Japan) at room temperature (≈22 °C), and the results were expressed as degrees Brix (Brix, Yountville, CA, USA). The color evaluation was determined as described in detail by Baibuch et al. [37]. The CIELab color space parameters were measured with a spectrophotometer (Minolta CM-5, Osaka, Japan) with a D_65_ illuminant and a 10° observer angle. The display was set to the CIELAB scale, with lightness (L*, relating to the luminosity between 0 (black) and 100 (white)), and two-color coordinates (a* and b*, varying from green (−) to red (+), and from blue (−) to yellow (+), respectively). In addition, the total color difference (ΔE^*^) (Equation (3)) values were calculated.

(3) $$\Delta E^{*}=\sqrt{{\left({\Delta L}^{*}\right)}^{2}+{\left({\Delta a}^{*}\right)}^{2}+{\left({\Delta b}^{*}\right)}^{2}}$$

where ΔL^*^ = (L^*^ − L^*^_0_), Δa^*^ = (a^*^ − a^*^_0_), Δb^*^ = (b^*^ − b^*^_0_). Subscript 0 indicates the color of the initial infusion, and L^*^, a^*^, and b^*^ indicate the color of the C_f_ in the respective cycle.

### 2.7. Determination of Total and Individual Bioactive Components

#### 2.7.1. Total Polyphenol Content (TPC)

TPC was determined according to the Folin–Ciocalteu method described by Yu et al. [38]. Thus, 100 μL of sample was mixed with 7.9 mL of distilled water, 500 μL of Folin–Ciocalteu reagent (2 M), and 1.5 mL of Na_2_CO_3_ (20% *w*/*v*). Then, the solution was vigorously mixed, and after 2 h of incubation in the dark at room temperature (≈22 °C), the absorbance was measured at 760 nm on a spectrophotometer (T70 UV/vis, Oasis Scientific Inc., Taylors, SC, USA). Gallic acid (GA) was used for the standard curve, and the TPC results were expressed as mg of GA equivalent (GAE) per mL of sample (mg GAE/mL).

#### 2.7.2. Total Flavonoid Content (TFC)

TFC was measured using the colorimetric method reported by Dewanto et al. [39]. Thus, 250 μL of sample was mixed with 1.25 mL of distilled water and 75 μL of NaNO_2_ (5% *w/v*). After 6 min, 15 mL of AlCl_3_ was added to the solution, and after 5 min, 500 μL of NaOH (1 M) and 275 μL of distilled water were added to the solution. Then, the absorbance was measured at 510 nm using a spectrophotometer (T70 UV/vis, Oasis Scientific Inc., Taylors, SC, USA). Catechin (C) was used for the standard curve, and the TFC results were expressed as mg of C equivalents (CE) per mL of sample (mg CE/mL).

#### 2.7.3. Polyphenolic Profile Using HPLC

The polyphenolic profile was characterized using the method described by Erdoğan and Erdemoğlu [40], with some modifications. Chromatographic separations were performed on an HPLC system (Shimadzu, LC-2050 C 3D, Kyoto, Japan) equipped with a UV detector, with a Nucleodur C18 Isis column (5 µm, 250 mm × 4.6 mm, Macherey-Nagel, Düren, Germany) with the column temperature at 25 °C. Gradient elution was performed using a mobile phase A, containing 3% of CH_3_;COOH in water, and a mobile phase B composed of CH_3_;COOH, CH_3_CN, and water (3:25:72 *v*/*v*). Sample elution followed the gradient program outlined in Table 2, with an injection volume of 20 mL, and detection was performed at different wavelengths between 280 and 355 nm. Compound identification relied on the comparison of retention time values and UV spectra with standards stored in a database. Polyphenolic profile concentrations were determined by calculating the integrated areas of the samples and corresponding standards. This method was previously validated to ensure its accuracy and reliability in polyphenolic analysis.

### 2.8. Determination of Antioxidant Capacity

#### 2.8.1. 2,2-Diphenyl-1-picrylhydrazyl (DPPH) Assay

The DPPH assay was performed using the method described by Brand-Williams et al. [41], with slight modifications. Thus, 150 μL of sample was mixed with 2.85 mL of DPPH methanolic solution (0.1 mM, where 10 mg DPPH was dissolved in 125 mL methanol). After 20 min in the dark at room temperature (incubation), the absorbance was measured at 515 nm using a spectrophotometer (T70 UV/vis, Oasis Scientific Inc., Taylors, SC, USA).

#### 2.8.2. Ferric Reducing Antioxidant Power (FRAP) Assay

The FRAP assay was conducted following the method outlined by Benzie and Strain [42], with some modifications. The FRAP reagent was prepared by combining 25 mL of CH_3_COONa (300 mM, pH = 3.6), 2.5 mL of TPTZ (10 mM in HCl (40 mM)), and 2.5 mL of FeCl_3_·6H_2_O (20 mM). Then, the mixture was incubated in the dark at 37 °C for 30 min. Subsequently, 150 μL of sample was mixed with 2.85 mL of the FRAP reagent. The solution was kept in the dark at 37 °C for 30 min, and the absorbance was measured at 593 nm using a spectrophotometer (T70 UV/vis, Oasis Scientific Inc., Taylors, SC, USA).

The DPPH and FRAP results were reported in μM Trolox equivalents (TE) per mL of sample (μM TE/mL).

### 2.9. Statistical Analysis

For the results, all measurements were made in triplicate, where the mean values were reported as mean ± standard deviation, with three replicates for each treatment. The data processing was executed using the Statgraphics Centurion XVI Software (v. 16.2.04, Statpoint Technologies Inc., Warrenton, VG, USA). A one-way analysis of variance (ANOVA) was carried out to recognize differences between samples, and a least significant difference test (LSD) test was conducted for the pairwise comparisons of means at a significance level of 5% (*p* ≤ 0.05).

## 3. Results and Discussion

### 3.1. Optimization of BFC Procedure by BBD

The separation parameters involved in the CBFC of peppermint infusion were optimized using the BBD, where a total of 15 experimental runs were designed and conducted according to the configured analysis. The BBD with coded variables and experimental and predicted data for the response is shown in Table 3.

Hence, the η in one cycle reached values between 65 and 81%, with runs conducted at intermediate centrifugation speed achieving values around 80%. These results are aligned with those found by Meneses et al. [29] in green tea (*Camellia sinensis*) extracts and Vásquez-Castillo et al. [43] in pomegranate juice. These authors reached an η of 70 to 80% in one cycle at centrifugation speed and time range between 1000 and 4600 rpm, and from 4 to 12 min, respectively. The high-efficiency values may be attributed to the low solids content in the concentrated solution, resulting in low viscosity, which facilitates the extraction of solutes through channels between the ice crystals by the centrifugal force.

A multiple regression analysis was applied on the experimental data. The predicted response variable and the independent variables were correlated, and a second-order polynomial model of η as a function of centrifugation conditions (speed (CS, X_1_), temperature (CT, X_2_), and time (Ct, X_3_)) was generated as shown in Equation (4):

(4) $$Y\;\left(\eta ,\;\%\right)=-34.2531+0.055425X_{1}+1.86X_{2}+0.02X_{3}{{\;-\;0.0000081875X}_{1}}^{2}+\;0.00012X_{1}X_{2}+0.0001575X_{1}X_{3}{{\;-\;0.0605X}_{2}}^{2}-\;0.026X_{2}X_{3}{{\;-\;0.006375X}_{3}}^{2}$$

A high agreement between experimental and predicted values for η was obtained by using the second-order polynomial equation, with a coefficient of determination (R^2^) value of 98.4%, where only 1.6% was not explained by the model. Therefore, the Equation (4) can be useful to predict the η under specific centrifugation experimental conditions.

Moreover, an ANOVA was performed to test the adequacy and fitness of the mathematical model. The regression coefficient values of the above equation obtained from the statistical analysis are shown in Table 4.

**Figure 2:**
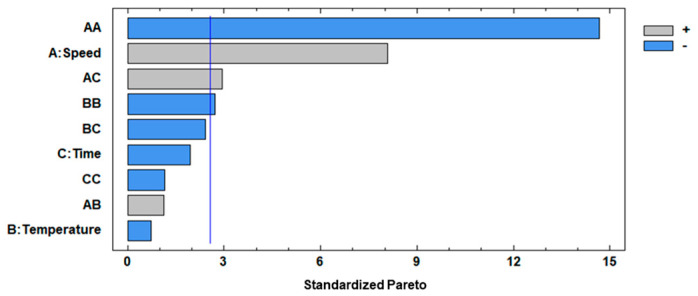
Standardized Pareto chart for η. A: speed; B: temperature; C: time.

Both the F test and *p*-value at a 95% confidence level were evaluated for analyzing the significance of the mathematical model, where the F-test values were higher than *p*-values, implicating a significant effect on the respective response variable. The factors with considerable influence on the separation step applied to the peppermint infusion were X_1_ (CS), X_1_^2^ (quadratic interaction of CS), X_1_X_3_ (interaction of CS and Ct), and X_2_^2^ (quadratic interaction of CT) which were considered significant, showing *p*-values < 0.05, with 0.0005, 0.0000, 0.0323, and 0.0422, respectively, and this indicates that these factors had effects on the response variable. The standardized Pareto (Figure 2) specified the influence of variables in the separation of C_f_ from I_f_ using different centrifugation conditions.

As was mentioned, significant interactions between factors were observed (*p*-value < 0.05), where X_1_ (linear term of CS) had positive effects on the separation process, meaning that an increase in its value led to the highest separation of C_f_ from I_f_, but X_1_^2^ (quadratic interaction of CS) had negative effects on the separation of C_f_ from I_f_, and this can be explained by the fact that a high centrifugation speed affects the extraction of solutes because as the CS increases, the ice crystals are more likely to break, resulting in smaller crystals. This size reduction can lead to greater tortuosity, potentially hindering the complete release of the concentrate [27]. For X_1_X_3_ (interaction of CS and Ct), it had a positive effect, meaning that an increase in both factors of separation allows a better extraction of fractions. However, a diffusion mechanism may occur at a higher CS, regardless of the time, causing the concentrate to return to the frozen fraction, resulting in low efficiency and solute recovery. The X_2_^2^ (quadratic interaction of CT) had negative effects on the separation step, and it can be explained by the fact that even a slight increase in temperature produces defrosting of the water, reducing the content of solids of C_f_ extracted from I_f_ [44]. Moreover, the optimum conditions proposed by BBD were identified as a CS of 3635.25 rpm, a CT of 16.00 °C, and a Ct of 13.84 min, and this combination allowed a maximum n of 81.51%. Hence, for operational convenience, the optimum conditions chosen were 3600 rpm, 16 °C, and 14 min for CS, CT, and Ct, respectively.

### 3.2. Response Surface Methodology

**Figure 3:**
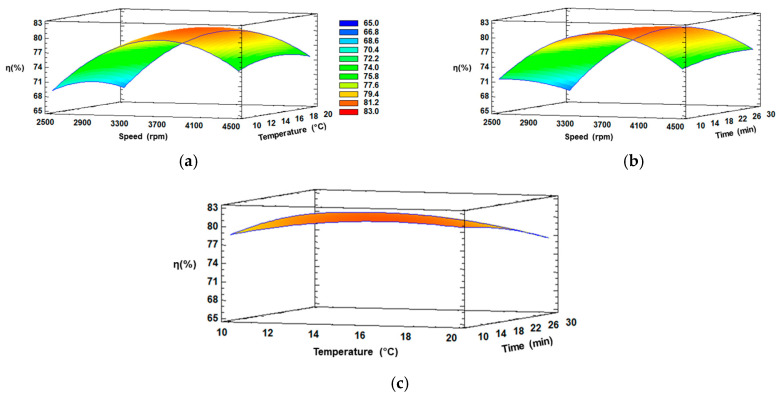
RSM plots of η (%) at interaction factors of: (**a**) CS and CT; (**b**) CS and Ct; (**c**) CT and Ct.

Three-dimensional response surface plots were generated using Statgraphics Centurion XVI Software version 16.2.04 to visualize the behavior of the independent variables (CS, CT and Ct), the dependent variable (η), and the interactions between these two (Figure 3).

Figure 3a,b illustrate the effect of CS and CT for 20 min, and CS and Ct at 15 °C, respectively. Similar behavior can be observed in both figures, since an increase in speed from 2500 to 3700 rpm with temperature from 10 to 16 °C or with time from 10 to 15 min, respectively, increased the extraction of C_f_ from I_f_, and later, the η decreased considerably at elevated speed (>3700 rpm) or longer temperature (>16 °C) or time (>15 min). The initial increase was due to the ice fraction remaining intact, whereby the C_f_ can be extracted without extracting water (in the ice phase), and thus, the final concentration is not affected by any dilution phenomenon.

On the other hand, a reverse trend could be observed with elevated speed (>3700 rpm) or longer temperature (>16 °C) or time (>15 min), and this can be explained since if the centrifugal conditions increase, they affect the ice structure, generating a rupture and dilution with the extract, which significantly reduces the concentration of solutes [45]. Thus, an excessive speed did not lead to an enhanced η. Meanwhile, in Figure 3c, the interaction between temperature and time were kept constant, with a slight increase in the intermediate temperature and minimum time, evidencing these factors not generate a significant separation of fractions, explained by the fact that an external force is necessary to improve the extraction of solutes from the channels of I_f_, since without an external force, the process is similar to gravitational-BFC, which is known for a low efficiency of separation [46]. Therefore, a positive equilibrium between speed and the other conditions can be achieved at approximately 3600–3700 rpm in the extraction of C_f_ from I_f_ through CBFC.

### 3.3. BFC Assisted by Centrifugation Procedure at Three Cycles

#### 3.3.1. Physicochemical Analysis

The physicochemical analysis results of the infusion and its extracts are shown in Table 5.

For TSC and TSSC, the infusion presented values close to 1.41% (*w/w*) and 1.81 °Brix, respectively, which are values significantly lower than those indicated by Dyab et al. [47], due to factors such as time of harvest; plant variety; edaphoclimatic conditions during plant growth such as water stress, soil composition, altitude, use of fertilizers, and temperature; industrial processing; storage conditions; type of packaging; and additionally, the part used in the extraction (leaves, aerials, and/or flowers), and type of extraction [48]. Thus, the application of CBFC generated an important increase in the TSC and TSSC content, with significant differences, in each extract obtained at each cycle. Hence, as cycles advanced, in the third cycle, TSC and TSSC values were close to 6.5% (*w*/*w*) and 8.7 °Brix, being 4.6 and 4.8 higher than the initial TSC and TSSC values, respectively. This significant rise in concentration is related to the freezing and separation conditions, since the combination of axial freezing at −20 °C allows a correct counter-diffusion phenomenon, and when the temperature decreased, the solutes were separated during the formation of ice crystals and located between the ice crystals, i.e., the solutes were expelled to the liquid fraction. Furthermore, the optimum conditions (CS, CT, and Ct) achieved a good elution phenomenon, and hence, at each cycle, a solution with considerable concentration can be extracted from the ice crystal channels [49]. A statistically significant rise in solute concentration has been observed in plant-based liquid matrices, since Boaventura et al. [28] obtained a final TSC value of 5.7% (*w*/*w*) after five gravitational-BFC cycles applied to mate extract, and similarly, Meneses et al. [29] reached a final TSC value close to 14.1% (*w*/*w*), with an initial value of 4.0% (*w*/*w*), after applying three gravitational-BFC cycles in green tea (*Camellia sinensis*) extract. Thereby, CBFC, with the freezing and optimal separation conditions, allows the obtaining of an extract with a considerable amount of solutes, and thus, there is a possibility to apply a greater number of cycles in future studies.

**Figure 4:**
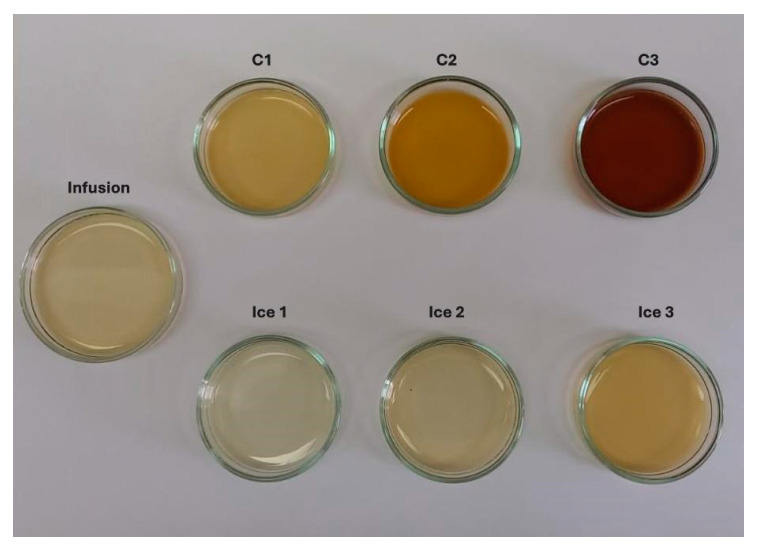
Changes in the color of infusion and extracts obtained by BFC technology.

In terms of color, the impact of CBFC on CIELab color parameters at three cycles is presented in Figure 4. As the cycles progressed, the L*, a*, and b* values decreased significantly compared to the initial infusion. The L* showed a value close to one, i.e., the final sample had an important loss of lightness (clarity). While, for infusion, a* and b* values indicated a bright green/yellow color, but, as cycles progressed, the values were closer to 0, and thus, it showed a darker brown color. These changes can be attributed to the increase in TSC and TSSC values and, in turn, to the decrease in the amount of water [50], intensifying the natural color of the infusion from cycle to cycle. The registered continual decline on CIELab color parameters has concordance with other liquid samples subjected to FC technology such as noni tea [30], pomegranate [49], arrayan, murta [51], strawberry juices [52], and coffee extract [53], where all the samples were darker with different behaviors in a* and b* values. More specifically, this behavior of extracts, with a clear decrease in L*, a*, and b*, has been observed when FC or thermal technologies were applied to concentrate a liquid food. Thus, a typical juice such as blueberry juice subjected to CBFC showed significant changes, where all the CIELab parameters decreased in comparison to the fresh juice [54]; similarly, pineapple juice showed the same behavior when concentrated in a thermostat water bath (95 °C for 3 min), with a decrease in the CIELab parameters, explained by the extraction of water and subsequent concentration of solutes, generating a darker sample that loses its original color [55].

During each cycle, an increase in the total color changes (ΔE*) was observed, and hence, for C_1_, the ΔE* was greater than 15 units, whereas C_3_ reached almost 95 units. According to Krapfenbauer et al. [56], a scale where ΔE* ≥ 3.5 CIELab units indicates that visual differences between two samples can be easily detected by the human eye. Therefore, the extracts can be differentiated from the infusion by human eyes. Other authors have reported similar results, obtaining ΔE* ≥ 3.0 units in concentrated samples after the FC process, with a darkening effect as the cycles increased [51,53].

#### 3.3.2. Total and Individual Bioactive Components

**Figure 5:**
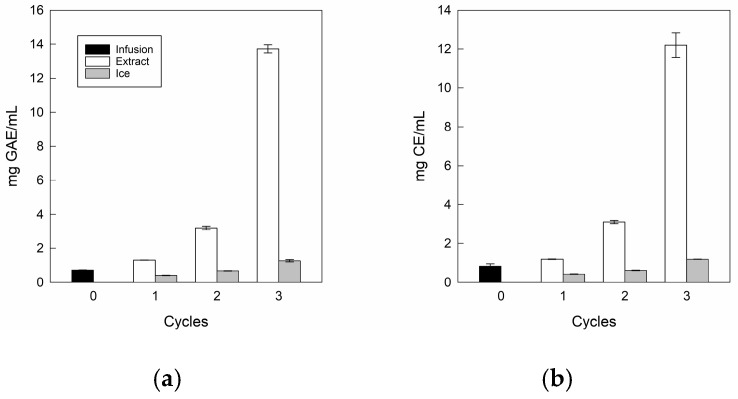
Total bioactive compound values of the infusion and extracts: (**a**) total polyphenol content; (**b**) total flavonoid content.

The total bioactive compounds in the infusion and its extracts at three CBFC cycles are depicted in Figure 5.

For TPC (Figure 5a) and TFC (Figure 5b), the infusion presented values close to 0.72 mg GAE/mL and 0.83 mg CE/mL, where our values were higher than those reported by Zielinski et al. [57] for *Mentha piperita* L. from Brazil, with 0.47 mg GAE/mL and 0.125 mg CE/mL, respectively. These differences could be due to the conditions of cultivation, maturity of the plant, time of harvest, plant variety, storage, infections, and edaphoclimatic conditions during its growth such as water stress, soil composition, altitude, and temperature, among others, influencing the formation of secondary metabolism [58].

For extracts, there are significant differences in both TPC and TFC values as the cycles progressed, reaching values close to 12.2 mg GAE/mL and 13.7 mg CE/mL, being approximately 17.0 and 16.5 times higher than the initial value, respectively. Notably, for the last cycle, in TPC terms, our results were 1.2 and 2.5 times higher than those achieved by Boaventura et al. [28] and Almeida et al. [31], who applied five CBFC cycles (10.34 mg GAE/mL) and four gravitational-BFC cycles (4.98 mg GAE/mL) in plant aqueous extracts such as aqueous extract of mate and noni tea, respectively. Whereas, in TFC terms, there are no studies to date on FC technology applied to plant-based liquid matrices that quantify flavonoids. However, our values were lower than those previously obtained in our laboratory using CBFC applied to apple juice [59]. Therefore, our conditions for separating phases allowed only three centrifugation cycles to achieve a high TPC and TFC content in the final extracts.

In comparable studies about FC technology, the bioactive compounds of pomegranate juice presented a significant increase with only one filtration using the CBFC (F-CBFC) method, increasing the initial total bioactive compound values for total anthocyanin and phenolic contents by 3.6 and 3.2 times, respectively, showing that it is possible to separate and concentrate water at an efficient rate from concentrated pomegranate juice [49]. While notably, when other concentration technology such as forward osmosis technology (27 °C) was applied to obtain extracts from rose petals, it showed a similar behavior to the FC method, since total anthocyanin content had a clear increase from 190 mg/L to 2130 mg/L, but the authors indicate that ideally a lower treatment temperature would help to further minimize the degradation of bioactive compounds [60].

Nevertheless, the ice fractions retained polyphenols and flavonoids as cycles advanced, since the residues from cycle 3 were higher than the initial values, with TPC and TFC values close to 1.27 mg GAE/mL and 1.19 mg CE/mL, respectively. This behavior, a continuous rise of total bioactive content in the ice fractions, can be attributed to the formation of hydrogen bonds with water molecules, causing some compounds to be trapped in water molecules and not move into the channels, and then, these are difficult to extract from the ice fraction [61]. Our results were consistent with the data published on FC technology, where the ice fraction showed greater bioactive compound content values as the cycle increased, indicating that many concentrated solutes are trapped between ice crystal channels cycle to cycle [62].

For the polyphenolic profile, 15 polyphenolic compounds were identified and quantified for the infusion and extracts, using the available standards (Table 6 and Supplementary Materials (Figure S1)).

For the initial sample, kaempferol and *p*-hydroxybenzoic were the major phenolic compounds identified, with concentration values close to 10.0 and 7.4 mg/L, respectively, followed by transferulic acid, myricetin, and caffeic acid, with a concentration of approximately 3.7, 2.8, and 2.7 mg/L, respectively. In addition, chlorogenic acid, protocatechuic acid, and ellagic acid were found in smaller quantities, with concentration values close to 0.9, 0.8, and 0.3 mg/L, respectively. In comparison to our findings, a study on *Mentha piperita* L. indicated that chlorogenic acid and rutin were the most abundant compounds [63], while another study with the same sample detected the highest concentrations in chlorogenic acid, caffeic acid, and kaempferol [64]. These different behaviors can be explained by the origin and growth of the food sample, influential environmental factors (weather conditions, soil, air, pluviosity, landforms, temperature, etc.), maturation status and agricultural practices (type/time) at harvest, and geographical characteristics (altitude), among others [65].

As cycles advanced, the concentration of phenolic compounds showed a significant upward trend, where in the last cycle, kaempferol, *p*-hydroxybenzoic, and transferulic acid exhibited the highest concentrations among the 15 polyphenolic compounds, with 177, 90, and 69 mg/L, respectively, and these concentration values were 18, 12, and 19 times higher than the initial concentration present in the infusion. The compounds with the lowest concentration (chlorogenic acid, protocatechuic acid, and ellagic acid) had a significant increase in concentration terms, since the concentration of compounds increased 5.1, 16.9, and 19.2 times in comparison to the initial concentration. These results align with those reported by several authors for applied FC technology for different food liquid samples such as noni tea [30,31], blueberry [62], maqui [66], and acerola pulp [67]. Hence, the significant increase in all compounds has been related to the increase in solids, allowing the increase of bioactive compounds, correlating with the individual compounds present in the liquid food sample. Thus, there is a direct correlation between solids, total bioactive compounds, and individual compounds, i.e., as one factor increases, it generates the increase of the other factors [68].

As a specific example, similar to the total bioactive components, when the FC method was applied to blueberry juice, a clear increase in the concentration of compounds could be visualized, where all the individual components were increased, demonstrating that FC technology retained many types of compounds due to the low temperatures of processing [61]. In turn, tomato juice processed by high-hydrostatic-pressure (550 MPa for 10 min at room temperature (approximately 25 °C)) showed a considerable concentration of its lycopene- and carotene-derived components; although, some biocomponents showed only a slight concentration (13-*cis*-*β*-carotene, 15-*cis*-*β*-carotene, 9-*cis*-*β*-carotene, and 15-*cis*-lycopene) [69], while FC technology has shown considerable increase in all individual biocomponents [70].

Based on this, the total and individual bioactive component results provide important information on the correct use of CS, CT, and Ct in the BFC process of concentrating any food solution, since it not only permits the extraction of concentrated solution from the ice fraction, but also there are considerable increases in the amount of solutes and the bioactive compound content, allowing the preservation of phenolic compounds though low temperatures. Thereby, the application of the CBFC process promotes a considerable recovery of different compounds in the concentrated fraction from different food matrices; however, studies have not yet used BFC as a concentration technology applied to endemic medicinal plants, allowing the extraction of biocomponents for medicinal, pharmacological, or food applications.

#### 3.3.3. Antioxidant Capacity of Infusion and Extracts

**Figure 6:**
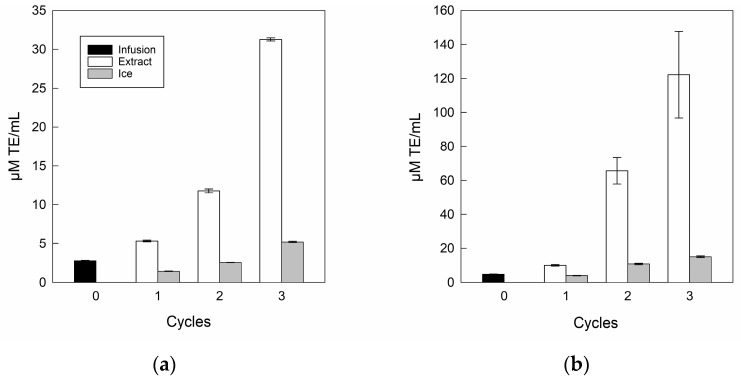
Antioxidant capacity of infusion and extracts: (**a**) DPPH; (**b**) FRAP.

The antioxidant capacities for the infusion and its extracts are shown in Figure 6.

In antioxidant capacity terms, the DPPH (Figure 6a) and FRAP (Figure 6b) assays showed a significant increase as the number of separation steps increased, where DPPH and FRAP values ranged from 2.8 to 31.2 μM TE/mL and 4.8 to 122.2 μM TE/mL, respectively, from the infusion to the last cycle, corroborating the upward relationship of bioactive compounds once the BFC process was used as the concentration technology with multi-separation steps [51]. Specifically, a similar trend was indicated by Zielinski et al. [71], who concentrated apple juice by BFC technology, with values from 1.3 to 5.5 μM TE/mL and 0.8 to 8.7 μM TE/mL for DPPH and FRAP, respectively, from the initial sample to the final cycle. However, the ice fractions retained antioxidant capacity in incremental cycles, whereas for the DPPH assay, only cycle 3 was higher than infusion, with a value close to 5.21 μM TE/mL. While, for FRAP assay, the residues from cycle 2 and cycle 3 were higher than the initial value, with values close to 10.82 μM TE/mL and 15.23 μM TE/mL, respectively. Hence, the increase in both DPPH and FRAP assays in the ice fractions can be attributed to the retention of biomolecules such as polyphenols and flavonoids between the ice crystals [45,50].

Additionally, in antioxidant capacity terms, many authors have indicated the advantage of FC technology applied to various food liquids such as noni tea [30,31], pomegranate [36], and prickly pear juice [72], where Abdelouhab et al. [73] explained that if the concentration of bioactive compounds increases, there is a significant increase in antioxidant capacity, i.e., there is a positive correlation between bioactive compounds and antioxidant capacity. Hence, the CBFC technology allows an interesting concentration of different bioactive compounds, and in turn, these increases in biocomponents can be reflected in the significant increase in antioxidant capacity.

## 4. Conclusions

This study evaluated the optimization and implementation of centrifugal-BFC technology to separate extracts of peppermint infusion from ice fractions and their evaluation in terms of physicochemical analysis, bioactive compounds, and antioxidant capacity, providing new information on the best conditions for obtaining phenolic-rich extracts through a novel, non-thermal technology. Thus, the results showed that the optimal centrifugation conditions for the separation of fractions were 3600 rpm, 16 °C, and 14 min, with an efficiency of separation close to 82%, the speed being the factor with the most significant influence with respect to the separation step. As cycles advanced, the best conditions allow a high content of solutes, total phenolic compounds, and antioxidant capacity. Moreover, the peppermint extracts had a good amount of individual bioactive compounds, highlighting kaempferol, *p*-hydroxybenzoic, and transferulic acid, and thus, these compounds could be candidates for cosmetic, pharmacological, biomaterial, and food applications, among others. However, it is very important to deepen research on some typical compounds of peppermint infusions and their incorporation into different food matrices, enhancing the nutritional and functional properties of various food formulations.

## Figures and Tables

**Table 1:** Box–Behnken design for surface analysis.

Factors ^1^	Code Units	Levels		
		−1	0	+1
CS (rpm)	X_1_	2500	3500	4500
CT (°C)	X_2_	10	15	20
Ct (min)	X_3_	10	20	30

^1^ CS: centrifugation speed; CT: centrifugation temperature; Ct: centrifugation time.

**Table 2:** Gradient program used for polyphenolic analysis by HPLC.

Analysis Time (min)	Eluent A	Eluent B	Flow Rate (mL/min)	Temperature (°C)
1–5	100	0	1	25
5–40 ^1^	30	70	1	25
40–45	20	80	1	25
45–55	15	85	1.2	25
55–57	10	90	1.2	25
57–75	10	90	1.2	25

^1^ For 5–40 min, the amount of solvent B was successively increased by 10% every 5 min.

**Table 3:** BBD design and results for the separation process of the C_f_ from the I_f_ in one cycle.

Run	Factors ^1^			η (%)	
	X_1_ (rpm)	X_2_ (°C)	X_3_ (min)	Experimental	Predicted
1	3500	20	30	76.8	76.6
2	2500	15	30	65.8	66.9
3	2500	15	10	71.4	71.5
4	4500	10	20	73.2	74.1
5	2500	10	20	69.5	69.2
6	2500	20	20	68.4	67.5
7	3500	10	10	78.5	78.7
8	3500	10	30	80.6	79.8
9	3500	20	10	79.9	80.7
10	4500	20	20	74.5	74.8
11	4500	15	10	75.6	74.5
12	4500	15	30	76.3	76.2
13	3500	15	20	81.1	81.1
14	3500	15	20	81.1	81.1
15	3500	15	20	81.1	81.1

^1^ X_1_: centrifugation speed; X_2_: centrifugation temperature; X_3_: centrifugation time.

**Table 4:** Estimated coefficients and significance test for linear, quadratic and interactive factors of the regression model.

Factors	Coefficient Estimate	Standard Error	F Value	*p*-Value	Significance ^1^
X_1_	6.13	0.76	65.27	0.0005	S
X_2_	−0.55	0.76	0.53	0.5007	NS
X_3_	−1.48	0.76	3.79	0.1093	NS
X_1_^2^	−16.38	1.12	215.32	0.0000	S
X_1_X_2_	1.2	1.07	1.25	0.3139	NS
X_1_X_3_	3.15	1.07	8.63	0.0323	S
X_2_^2^	−3.03	1.12	7.35	0.0422	S
X_2_X_3_	−2.60	1.07	5.88	0.0597	NS
X_3_^2^	−1.28	1.12	1.31	0.3050	NS

^1^ S = Significant, NS = Non-significant.

**Table 5:** Physicochemical properties of infusion and extracts obtained by the centrifugal-BFC process at three cycles.

Sample	TSC (% *w*/*w*)	TSSC (°Brix)	L*	a*	b*	ΔE*
Infusion	1.41 ± 0.06 ^a^	1.81 ± 0.00 ^a^	64.26 ± 2.13 ^a^	15.30 ± 1.97 ^a^	70.72 ± 0.49 ^a^	-
C1	1.96 ± 0.19 ^b^	2.53 ± 0.15 ^b^	50.58 ± 0.37 ^b^	26.92 ± 0.27 ^b^	76.81 ± 0.04 ^b^	18.97 ± 3.24 ^a^
C2	2.89 ± 0.24 ^c^	3.71 ± 0.71 ^c^	24.38 ± 3.56 ^c^	34.60 ± 0.44 ^c^	44.99 ± 2.67 ^c^	51.30 ± 5.57 ^b^
C3	6.50 ± 0.08 ^d^	8.69 ± 1.01 ^d^	1.10 ± 0.72 ^d^	0.28 ± 0.74 ^d^	1.05 ± 0.74 ^d^	94.97 ± 1.99 ^c^

Means ± standard deviation in the same row followed by different lowercase letters indicate statistically significant differences at *p* ≤ 0.05 for each sample (n = 3). TSC: total solid content; TSSC: total soluble solids content; L*: luminosity; a*: variation from green (−) to red (+); b*: variation from blue (−) to yellow (+); ΔE*: total color difference.

**Table 6:** Phenolic compounds identified in infusion and extracts using HPLC.

Phenolic Compounds (mg/L)	Feed Extract	C1	C2	C3
Chlorogenic acid	0.87 ± 0.00 ^a^	0.73 ± 0.66 ^a^	1.16 ± 0.10 ^a^	4.41 ± 0.22 ^b^
Gallic acid	ND	1.25 ± 0.42 ^a^	4.17 ± 0.42 ^b^	15.26 ± 5.48 ^c^
Protocatechuic acid	0.77 ± 0.25 ^a^	1.05 ± 0.01 ^b^	3.87 ± 0.39 ^c^	12.98 ± 0.45 ^d^
Catechin	1.35 ± 0.81 ^a^	1.71 ±0.44 ^a,b^	4.01 ± 1.70 ^c^	18.26 ± 6.16 ^d^
Caffeic acid	2.67 ± 0.11 ^a^	4.54 ± 1.06 ^b^	11.70 ± 1.22 ^c^	43.88 ± 1.25 ^d^
Vanillic acid	2.20 ± 1.38 ^a^	2.70 ± 0.05 ^a^	7.44 ± 0.83 ^b^	30.91 ± 1.25 ^c^
Syringic acid	1.40 ± 0.03 ^a^	2.40 ± 0.15 ^b^	5.87 ± 0.89 ^c^	29.46 ± 1.11 ^d^
*p*-coumaric acid	2.28 ± 0.12 ^a^	4.97 ± 1.73 ^b^	12.87 ± 2.81 ^c^	40.82 ± 0.92 ^d^
Ellagic acid	0.26 ± 0.05 ^a^	0.40 ± 0.00 ^b^	1.18 ± 0.11 ^c^	5.00 ± 0.32 ^d^
Transferulic acid	3.68 ± 0.14 ^a^	5.93 ± 0.12 ^b^	17.06 ± 2.09 ^c^	68.68 ± 1.08 ^d^
Rutin	1.65 ± 0.21 ^a^	3.18 ± 0.13 ^b^	15.27 ± 10.91 ^c^	28.82 ± 6.64 ^c,d^
*p*-hydroxybenzoic acid	7.36 ± 0.05 ^a^	11.87 ± 0.62 ^b^	39.66 ± 4.60 ^c^	89.95 ± 0.07 ^d^
Myricetin	2.82 ± 2.58 ^a^	2.61 ± 1.99 ^a^	18.19 ± 5.08 ^b^	45.31 ± 7.14 ^c^
Quercetin	1.00 ± 0.06 ^a^	1.14 ± 0.37 ^a^	3.83 ± 0.34 ^b^	16.04 ± 0.09 ^c^
Kaempferol	10.04 ± 0.68 ^a^	18.55 ± 1.20 ^b^	58.99 ± 12.60 ^c^	177.40 ± 4.57 ^d^

Means ± standard deviation in the same row followed by different lowercase letters indicate statistically significant differences at *p* ≤ 0.05 for each sample (n = 3).

## Data Availability

The data presented in this study are available on request from the corresponding author.

## Supplementary Materials

The following supporting information can be downloaded at: https://www.mdpi.com/article/10.3390/antiox14020129/s1. Figure S1: Chromatograms of samples: (a) Infusion; (b) Cycle 1; (c) Cycle 2; (d) Cycle 3.
