# Supplementary material for: Effect of Block Freeze Concentration on Bioactive Compound Content and Antioxidant Capacity When Applied to Peppermint (Mentha Piperita L.) Infusion
Source: Antioxidants (Basel). 2025 Jan 23;14(2):129. doi: 10.3390/antiox14020129 (PMC11851875; doi:10.3390/antiox14020129)
Supplement: Supplementary file 1 [file Supplementary Materials.pdf › Supplementary Materials.pdf]

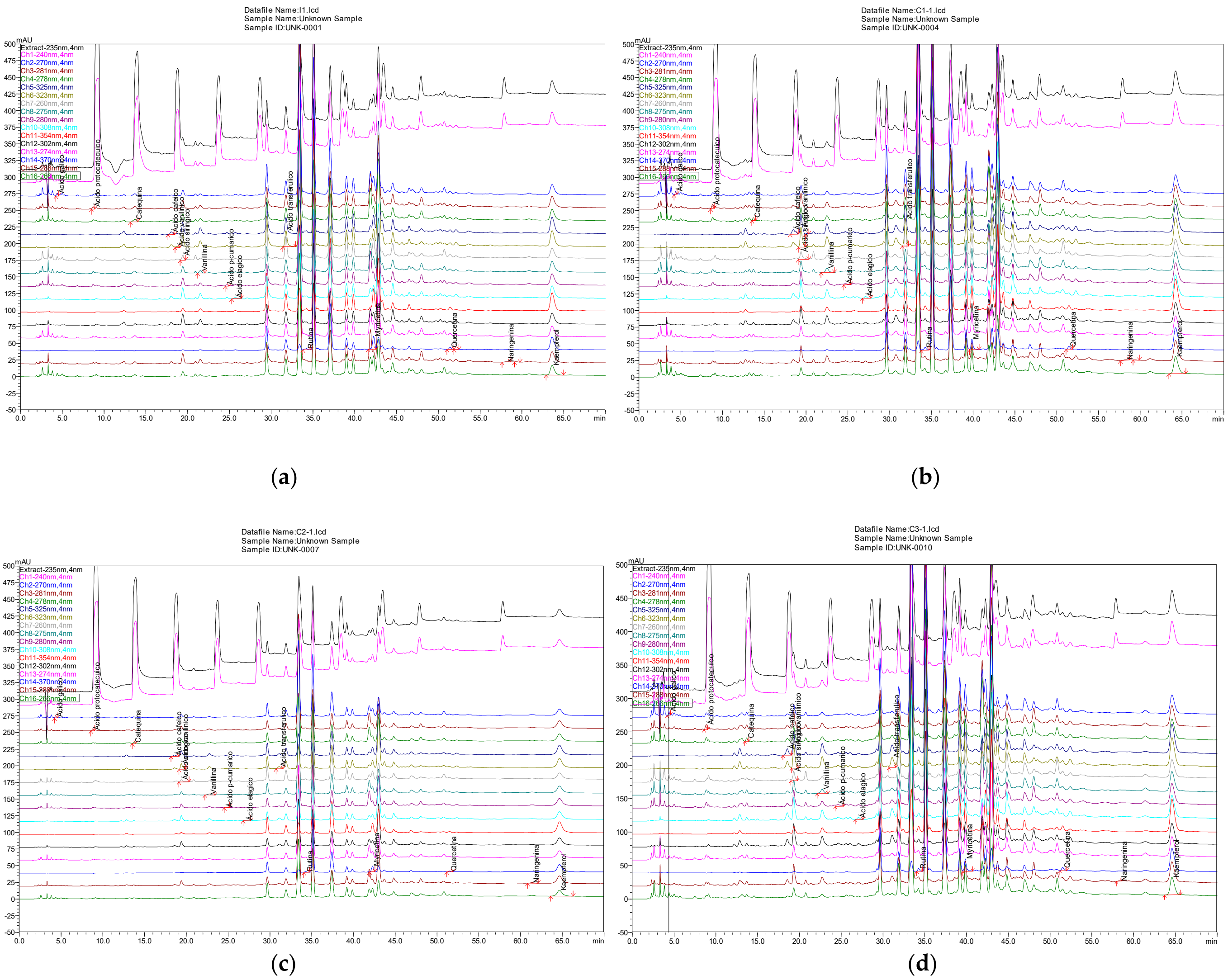


**Figure S1.** Chromatograms of samples: (**a**) Infusion; (**b**) Cycle 1; (**c**) Cycle 2; (**d**) Cycle 3.
